# Three-dimensional analysis of single molecule FISH in human colon organoids

**DOI:** 10.1242/bio.042812

**Published:** 2019-07-30

**Authors:** Manja Omerzu, Nicola Fenderico, Buys de Barbanson, Joep Sprangers, Jeroen de Ridder, Madelon M. Maurice

**Affiliations:** 1 Oncode Institute and Department of Cell Biology, Centre for Molecular Medicine, University Medical Centre Utrecht, 3584 CX Utrecht, The Netherlands; 2 Oncode Institute and Department of Genetics, Center for Molecular Medicine, University Medical Center Utrecht, 3584 CG, Utrecht, The Netherlands; 3 Oncode Institute, Hubrecht Institute, Royal Netherlands Academy of Arts and Sciences (KNAW) and University Medical Center Utrecht, 3584 CT Utrecht, The Netherlands

**Keywords:** Differentiation, Human organoids, SmFISH, Stem cells, Transcription

## Abstract

The culturing of mini-organs (organoids) in three-dimensions (3D) presents a simple and powerful tool to investigate the principles underlying human organ development and tissue self-organization in both healthy and diseased states. Applications of single molecule analysis are highly informative for a comprehensive understanding of the complexity underlying tissue and organ physiology. To fully exploit the potential of single molecule technologies, the adjustment of protocols and tools to 3D tissue culture is required. Single molecule RNA fluorescence *in situ* hybridization (smFISH) is a robust technique for visualizing and quantifying individual transcripts. In addition, smFISH can be employed to study splice variants, fusion transcripts as well as transcripts of multiple genes at the same time. Here, we develop a 3-day protocol and validation method to perform smFISH in 3D in whole human organoids. We provide a number of applications to exemplify the diverse possibilities for the simultaneous detection of distinct mRNA transcripts, evaluation of their spatial distribution and the identification of divergent cell lineages in 3D in organoids.

## Abstract (precis)

**Summary:** We present a sensitive single-molecule (sm)FISH application for the identification of individual mRNA transcripts and their spatial distribution in 3D in whole human colon organoids.

## INTRODUCTION

Over the last decade, considerable advances have been made in the development of 3D tissue culture systems (Clevers, 2016). In the first reported long-term cultured organoid model, Lgr5-positive (Lgr5^+^) intestinal stem cells (ISC) were isolated from murine small intestinal crypts (Sato et al., 2009). When subjected to optimized growth conditions, ISCs and their progenitors exhibited self-organization *in vitro* into intestinal crypt–villus structures that contained all different cell types found in the small intestinal epithelium *in vivo*. Further improvement of culture protocols allowed for the establishment of organoids derived from human healthy colon stem cells and their cancer-derived equivalents as well (Jung et al., 2011; Sato et al., 2011). Since then, human colon organoids have been employed in several applications, including disease modelling, the generation of colorectal cancer biobanks, as well as predictors for personalized medicine applications (Dekkers et al., 2016; Drost and Clevers, 2017; Drost et al., 2015; Matano et al., 2015; van de Wetering et al., 2015). In addition, organoids were used to portray individual cell types in their physiological context as well as during disease onset and progression (Clevers, 2016; Huch and Koo, 2015). Moreover, the modulation of key signaling cascades within organoids has enabled mechanistic insights into the regulation of cell plasticity, lineage specification and the formation of rare cell types (Basak et al., 2017; Drost and Clevers, 2017; Jung et al., 2011; Sato et al., 2011).

Single molecule RNA fluorescence *in situ* hybridization (smFISH) allows for the visualization of individual RNA molecules by employing a pool of short, singly labelled oligonucleotides that selectively recognize their RNA target by the formation of Watson-Crick base pairs (Femino et al., 1998; Lyubimova et al., 2013; Raj et al., 2008). smFISH-based mRNA visualization was applied successfully to multiple model systems, including yeast (Rahman and Zenklusen, 2013), nematodes (Ji and van Oudenaarden, 2012), fruit fly (Bayer et al., 2015; Trcek et al., 2017), mammalian cells (Buxbaum et al., 2015; Raj et al., 2008) and tissues (Lyubimova et al., 2013; Raj et al., 2008). smFISH applications have been instrumental in the detection and analysis of rare cell types, fusion transcripts and splice variants and, importantly, allows for automated transcript quantification combined with spatiotemporal information (Crosetto et al., 2015).

Thus far, studies that applied smFISH to mammalian tissues and organoids involved the use of thin tissue sections, thereby losing valuable 3D information on the location and positioning of individual cells within the tissue (Lyubimova et al., 2013; Schuijers et al., 2015). Hence, preserving 3D organoid architecture would greatly advance the usage of smFISH applications in the evaluation of single cell behavior within their pertinent tissue context. Here, we describe a protocol for the application and analysis of 3D smFISH in whole human organoids. We describe key optimization steps for sample preparation and provide a method for the analysis of 3D smFISH sensitivity and specificity. Finally, we demonstrate a number of smFISH applications for the spatial and quantitative evaluation of cell signaling responses and transcriptional alterations during cell lineage specification in organoids. Together, our method provides a robust contemporary tool that can be applied easily in standard equipped laboratories.

## RESULTS AND DISCUSSION

### Optimization of sample preparation

Organoid-based methods for understanding tissue homeostasis and pathology continue to expand (Clevers, 2016; Rossi et al., 2018), but a detailed protocol for the 3D assessment of *in situ* hybridization in organoids has not been described. We set out to develop a stringent and reliable method for the spatial evaluation of individual mRNA transcripts in 3D tissue cultures that can be implemented easily in labs equipped with standard wide-field and confocal microscopes. The workflow is based upon a previously established protocol (Lyubimova et al., 2013) that consists of three steps: sample preparation, *in situ* hybridization and image acquisition and quantification.

**Fig. 1.**
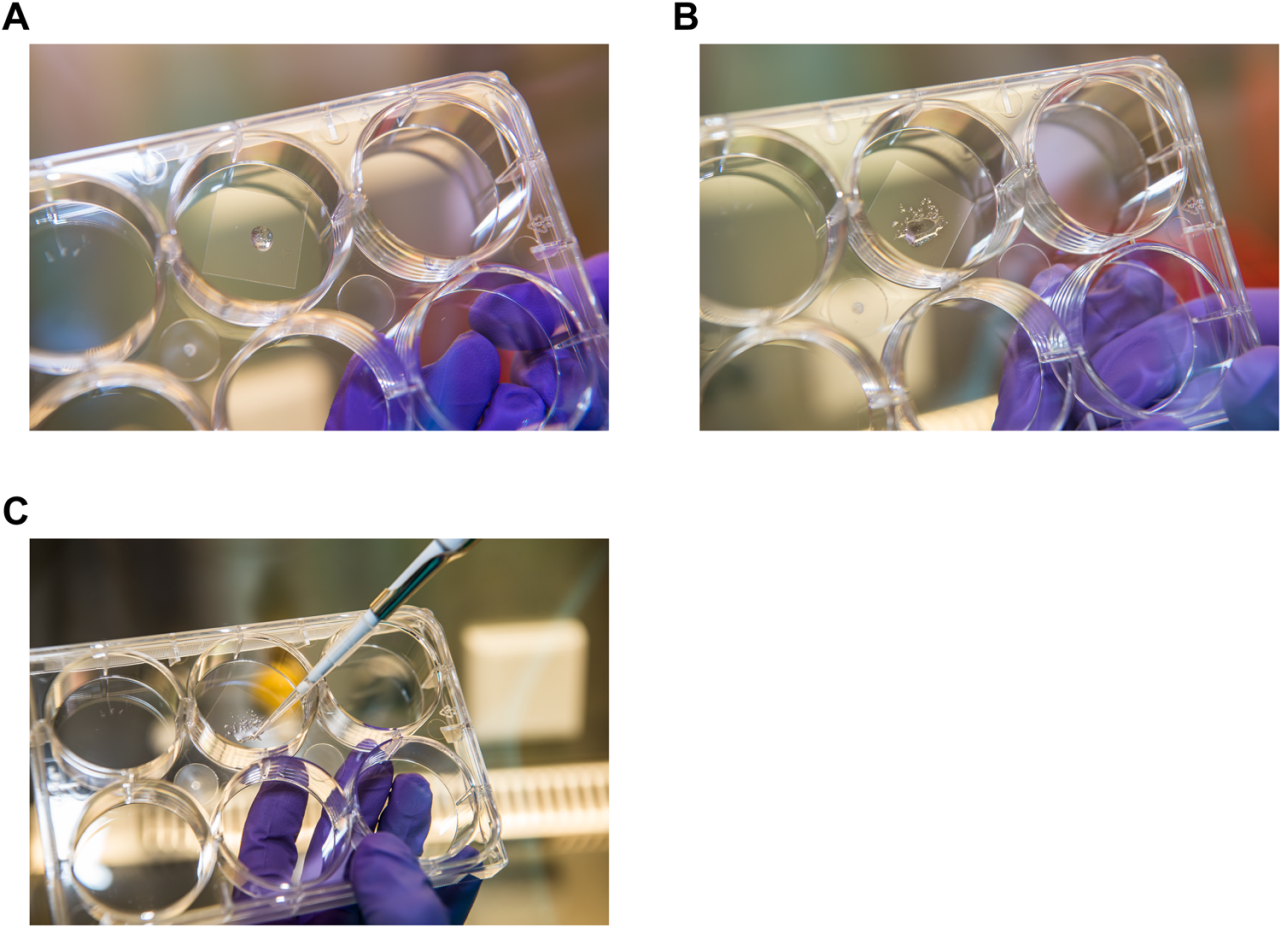
**Optimized protocol for smFISH on whole human organoids.** (A) Matrigel-free organoids were re-suspended in 10 µl of RNAse free PBS and spotted as a droplet on poly-K coated coverslips. (B) The droplet was distributed over a bigger area using a pipet tip. (C) Residual PBS was removed.

To preserve 3D architectural structure, human colon epithelial organoids are embedded in extracellular matrix-derived gels (e.g. Matrigel). Such gel materials generally carry strong auto-fluorescent properties, compromising the application of fluorescence-based techniques (Kim et al., 2015). We thus aimed to effectively remove Matrigel while preserving 3D architecture, to obtain an optimal signal-to-noise ratio in fluorescent ISH applications. To this end, we seeded organoids in a reduced Matrigel percentage (Matrigel to medium ratio of 1:1) 48 h before applying smFISH. Organoid growth and morphology remained unaffected by these conditions when compared with standard culture conditions (Fig. S1A). In addition, before harvesting we treated organoids with Cell Recovery Solution, a Matrigel-depolymerizing reagent compatible with subsequent protein or RNA analysis (Matano et al., 2015). Optimization of this step is described below. After washing, Matrigel-free organoids were spotted as droplets on coverslips (Fig. 1A–C, see Materials and Methods). This part of the protocol can be employed for the analysis of organoids by conventional immunofluorescence as well.

### Employing fluorescence co-localization in 3D for smFISH validation

**Fig. 2.**
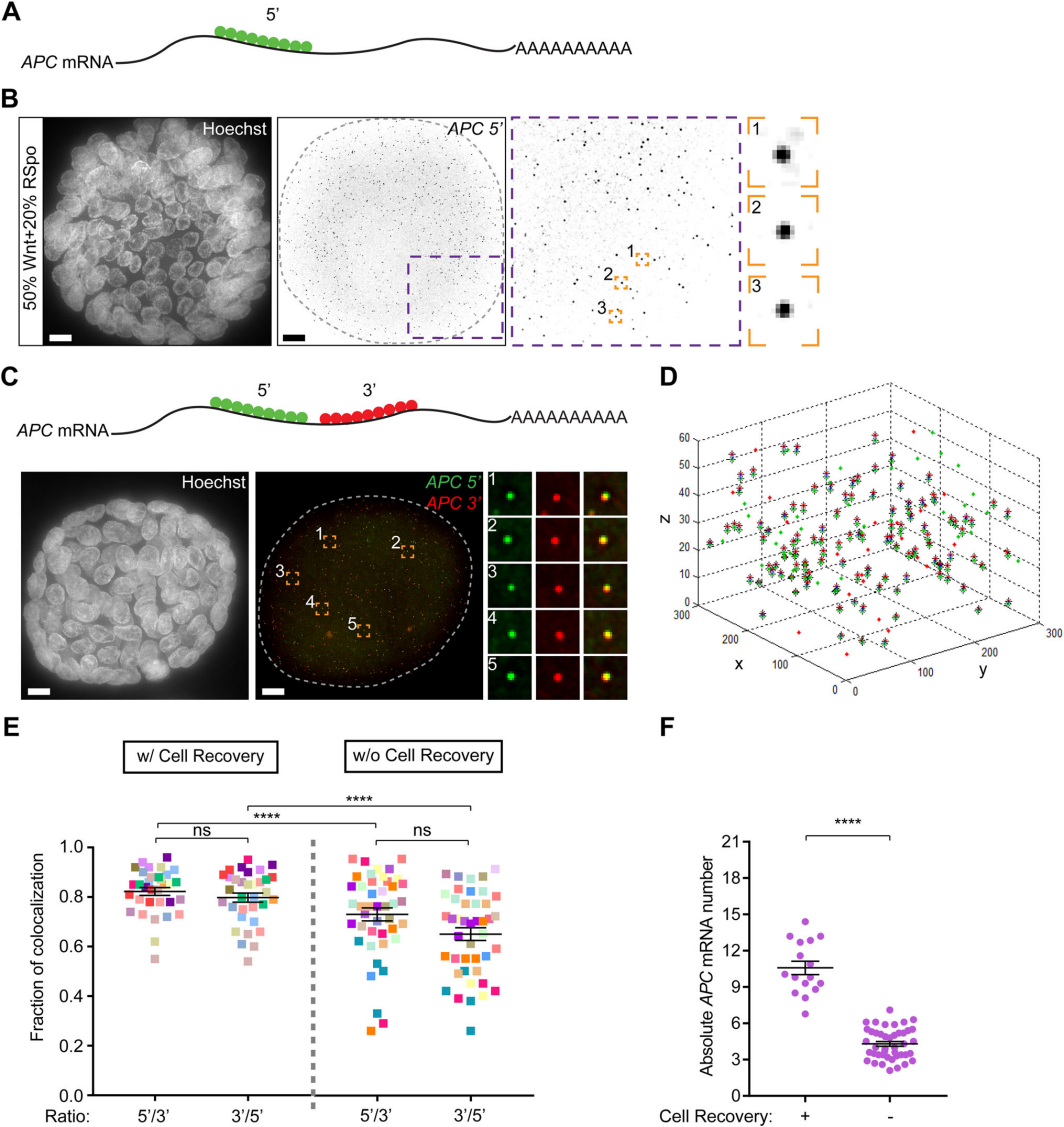
**Using fluorescence co-localization for validation of 3D smFISH sensitivity and specificity.** (A) Schematic of smFISH probes targeting the coding region of *APC* mRNA. A pool of 48 fluorescently labeled probes (Quasar 670) targeting the 5′ end of the *APC* transcript was designed and produced by Stellaris. (B) smFISH signal appears as a bright fluorescent dot (indicated as black dots in the figure) against a uniformly white background. Hoechst-based staining of nuclei is shown as a reference for the localization of individual colon organoid cells. The boundary of the organoid in the middle panel is marked with a dashed line. Insets show magnification of distinct organoid region (purple) and individual mRNA dots (orange). Individual dots and corresponding panels are indicated in numerical order. Scale bars: 10 μm. (C) Schematic representation of two *APC* mRNA smFISH probe sets targeting a distinct *APC* coding region (Quasar 670 and TAMRA). The boundary of the organoid in the middle panel is marked with a dashed line. Insets show magnification of individual mRNA dots and their co-localization. Individual dots and corresponding panels are indicated in numerical order. (D) Graph represents 3D localization of transcripts. Red spots in the graph correspond to 3′ *APC* probes, while green spots correspond to 5′ *APC* probes. Paired co-localizing dots are marked with crosses. Pixel coordinates are plotted on x and y axis, z axis shows index of z-stack. (E) Ratio of *APC* mRNA co-localization between the two probe sets in organoids treated with (w/; *n*=13) or without (w/o; *n*=14) Cell Recovery solution. Organoids are color-coded, each color indicates ROI within a single organoid. Data of three independent experiments are shown here (±s.e.m.). To test significance, we used ordinary one-way ANOVA. Ns, not significant. (F) Matrigel removal allows detection of an overall higher number of mRNA particles compared to non-treated organoids. Single dots represent average mRNA numbers per cell, as determined by mRNA count in different ROIs. Hoechst stain was used for cell number determination. Treated sample, *n*=16 cells; non-treated sample, *n*=43 cells. Data of three independent experiments are shown here (±s.e.m.). To test significance, we used unpaired *t*-test. ****P<0.0001.

Once organoids were spotted on coverslips, we continued with hybridization. For evaluation of smFISH specificity, we targeted the transcript of *Adenomatous Polyposis Coli* (*APC*), a key Wnt pathway tumor suppressor that plays well-established roles in the maintenance of colon tissue homeostasis (van Kappel and Maurice, 2017). We made use of a singly labelled probe set consisting of a pool of 48 oligonucleotides that target the coding region of the *APC* mRNA (Fig. 2A). We observed bright fluorescent spots that were easily visualized in both the cytosol and nucleus of organoid cells, indicating the detection of individual *APC* mRNA species (Fig. 2B). mRNA particles in organoids were subjected to automated spot detection using a MATLAB code (Raj et al., 2008) in which particles are segmented from 3D images. The resulting mRNA dot volumes show a unimodal distribution (Fig. S1B). In addition, we confirmed that mRNA dots displayed a linear correlation between volume and intensity of dots in one channel after applying the threshold (Fig. S1C, see Materials and Methods).

In order to verify the specificity of the obtained signals we designed a second set of *APC* probes that target a distinct region of the same transcript, located towards the 3′ end of the mature *APC* mRNA. We labelled this second probe set with a different fluorophore to determine co-localization of both probe sets as a stringent measurement of smFISH specificity for a single transcript (Fig. 2C). To control for probe specificity in each channel, we measured co-localization efficiency of 5′ versus 3′ and 3′ versus 5′ probes. This set-up was first validated in SW480, a human colorectal epithelial cell line, where we verified the specificity of our *APC* smFISH probe sets by manipulating endogenous *APC* transcription. Specifically, we employed an RNA guided nuclease-null Cas9 (dCas9) variant fused to either VP64-p65-Rta (VPR) tripartite activator to increase transcription (Chavez et al., 2015) or to the Krüppel-associated box (KRAB) repressor domain to suppress transcription (Thakore et al., 2015) of the *APC* gene. Analysis of untreated SW480 cells showed equal levels of co-localization of *APC* 5′ with 3′ probes as well as 3′ with 5′ probes (87 and 88%, respectively; Fig. S1D), indicating that both probe sets displayed similar levels of specificity. Of note, co-localizing fractions were similar in conditions where the total number of transcribed *APC* mRNA levels were either strongly increased or decreased (Fig. S1D,E). These results confirm the high specificity of this method in 2D cell culture across a range of mRNA levels. Co-localization levels are in line with a previous study in which two sets of probes were used to target a single transcript (Raj et al., 2008).

Next, we employed 5′- and 3′-targeting RNA probe sets to address smFISH specificity in organoids treated with or without Cell Recovery Solution. For hybridization, a previously described protocol was used (Lyubimova et al., 2013). Organoid images were taken by collecting z-stacks (spanning around 18 μm) and analysis was performed on an average of three regions of interest (ROI, 300×300 pixels) per organoid, covering distinct segments of the 3D structure. The analysis proposed in Raj et al. (2008) was optimized by including a pairwise Euclidian distance calculation between spots in two channels to allow for automated detection of fluorescence co-localization in 3D (Fig. 2D, see Materials and Methods). This approach avoids data underestimation by single plane image quantification and decreases the probability of falsely paired dots as compared to maximum-projected images. Organoids not treated with Cell Recovery Solution revealed strong variability in signals between channels with a greater proportion of unpaired probes, signifying suboptimal detection of specific *APC* mRNA species under these conditions. In addition, the number of detected mRNA particles per cell was rather low, suggesting that remnants of Matrigel might prevent reliable probe penetration and/or smFISH signal detection (Fig. 2E,F). Indeed, treatment with Cell Recovery Solution allowed for a significantly higher degree of co-localization between both probe sets in several organoids that were analyzed, confirming reliable transcript detection (Fig. 2E). Of note, longer treatments (60 min) with Cell Recovery decreased smFISH signal specificity, therefore we limited incubation times to a maximum of 15 min (Fig. S1F).

### Monitoring transcriptional alterations in human colon organoids using smFISH

Next, we investigated the sensitivity of our smFISH method for the detection of signaling-induced alterations in transcriptional activity in 3D. To this end, we subjected human colon epithelial organoids to variable concentrations of Wnt3a, an essential growth factor for stem cell maintenance and cell specification (Jung et al., 2011; Sato et al., 2011). As endogenous expression of Wnts in human colon organoids is low, supplementation with Wnt3a and the Wnt-potentiating factor R-spondin is required for long-term propagation of such tissue cultures (Jung et al., 2011; Sato et al., 2011). Consequently, withdrawal of Wnt and R-spondin induces a loss of stem cell gene expression and the induction of a transcriptional program for cellular differentiation (Lindeboom et al., 2018; Sato et al., 2011). To investigate the possibility of applying smFISH for monitoring alterations in Wnt-induced transcription, we focused on the expression of *AXIN2*, a well-defined and universal Wnt target gene (Jho et al., 2002; Yan et al., 2001).

**Fig. 3.**
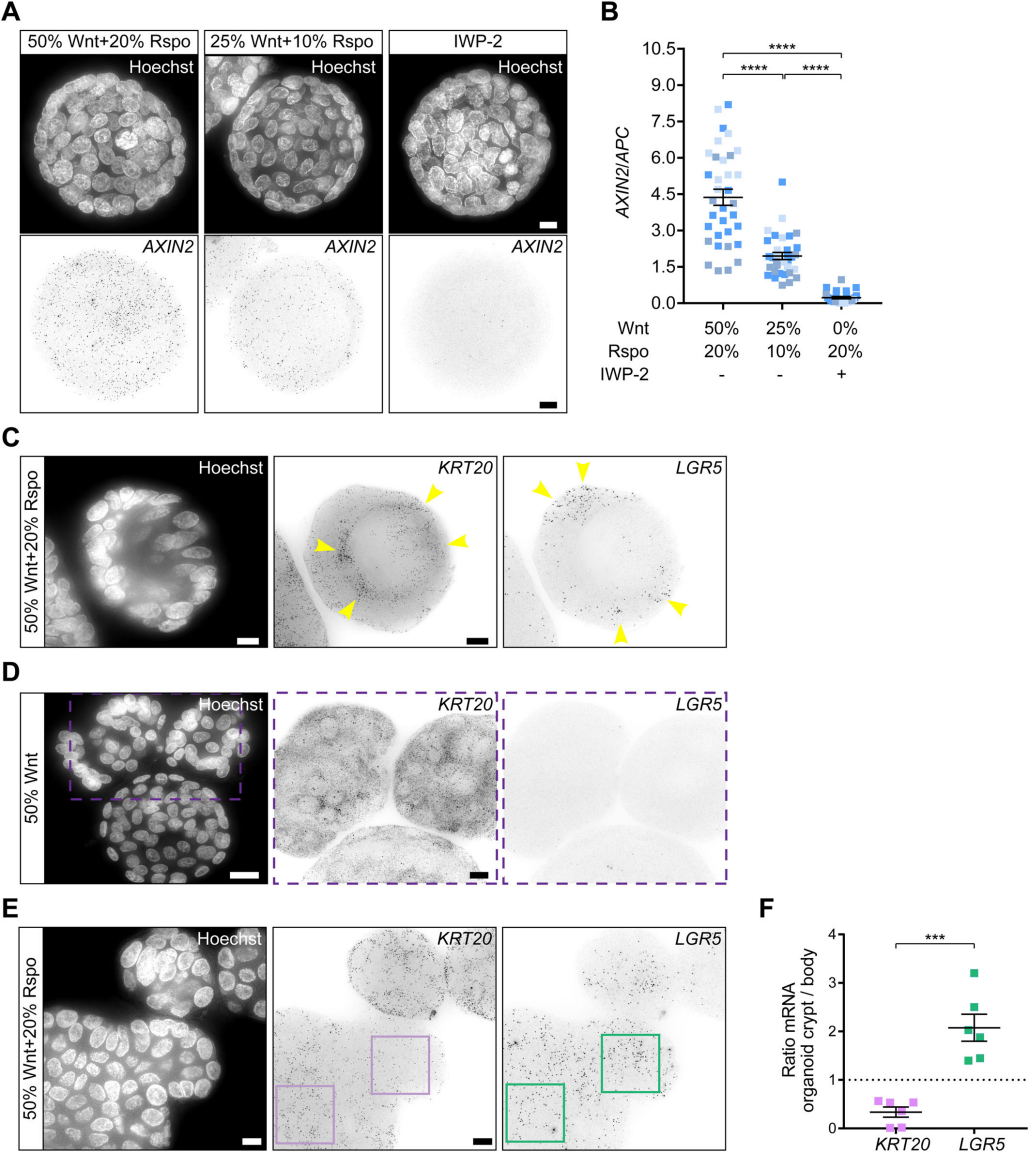
**Monitoring signal-induced transcriptional alterations using 3D smFISH in human colon organoids.** (A) Expression analysis of *AXIN2* (Quasar 670) upon Wnt pathway modulation. Black dots represent the *AXIN2* smFISH signal. Hoechst-based staining of nuclei is shown as a reference for the localization of individual cells. Organoids were grown in different amounts of Wnt3a and R-spondin (Rspo) or were treated with IWP-2 as indicated. Scale bars: 10 μm. (B) Quantification of *AXIN2* mRNA expression in organoids, grown in different medium. *AXIN2* mRNA number is normalized to *APC* mRNA number. One square in the graph represents one ROI. Full medium, *n*=19; Wnt3a and R-spondin reduced *n*=18; IWP-2 *n*=16. Data of three independent experiments are shown (±s.e.m.). To test significance, we used ordinary one-way ANOVA. ****P<0.0001. (C) Human colon organoids grown in standard medium for 2 days show anti-correlated expression patterns of stem cell marker *LGR5* (Quasar 670) and pan-differentiated marker *KRT20* (Quasar 570). Arrowheads indicate the transcript enrichment localization. Hoechst is used for nuclei staining. Scale bars: 10 μm. (D) R-spondin withdrawal causes downregulation of *LGR5* and a strong increase of *KRT20* expression. Hoechst is used for nuclei staining. Scale bars: 10 μm. (E) smFISH analysis of ‘budding’ organoid displays differential *LGR5* and *KRT20* expression patterns in stem-cell enriched crypt versus the body of the organoid. The squares indicate ROI that was taken for quantification. Hoechst is used for nuclei staining. Scale bars: 10 μm. (F) Quantification of *LGR5* and *KRT20* mRNA in two distinct ROIs of ‘budding’ organoid. Graph displays a ratio between absolute mRNA numbers detected in organoid body and the crypt. Analyzed organoids *n*=6. Data of three independent experiments are shown (±s.e.m.).To test significance, unpaired *t*-test was used. ***P=0.0002.

Downregulated Wnt pathway activity in human colon organoids by supplementation with decreased levels of Wnt3a and R-spondin for 48 h, induced a clear decrease in *AXIN2* smFISH signals. Furthermore, treatment with 2.5 μM of Wnt secretion inhibitor IWP-2 (Chen et al., 2009) for 48 h mediated a drop in *AXIN2* smFISH signals to largely undetectable levels (Fig. 3A). Of note, *APC* smFISH signals remained unaltered under these conditions (Fig. 3B; Fig. S2A), indicating that the loss of *AXIN2* signals was not due to an overall increase in cell death. To validate reliability of the quantification of mRNA signals, we additionally performed RT-qPCR analysis of organoids treated with decreased levels of Wnt3a/R-spondin or IWP-2. The results confirm decreased *AXIN2* levels when Wnt levels are depleted, in line with our smFISH data (Fig. S2B). These results show that our smFISH method enables reliable and sensitive quantification of endogenous transcript alterations in organoids.

### Identifying and localizing distinct cellular identities in organoids using smFISH

Next, we aimed to validate the application of our smFISH method for the detection of different cellular identities that are spatially distributed in 3D. We exploited the strict dependency of LGR5^+^ stem cells on the combined stimulation with Wnt and R-spondin signals (Yan et al., 2017). Both factors are essential for the formation and maintenance of specialized stem-cell enriched domains and the propagation of human colon organoid cultures (Sato et al., 2011). Differentiated cells in the intestinal epithelium are recognized collectively by the marker keratin-20 (KRT20) (Chan et al., 2009). Organoids grown in full medium (50% Wnt3a-CM and 20% Rspo1-CM) displayed a characteristic cystic morphology and revealed the existence of distinct domains positive for either *LGR5* or *KRT20* smFISH (Fig. 3C). Depletion of R-spondin reduces the levels of Wnt signaling and drives collective differentiation at the expense of stem cell populations (Yan et al., 2017). As expected, we observed a complete loss of *LGR5* smFISH signals and a significant increase in the expression of *KRT20* upon withdrawal of R-spondin for 48 h (Fig. 3D).

Finally, we employed our 3D smFISH method to spatially characterize intra-organoid heterogeneity. Human colon organoids can spontaneously assemble budding crypt structures. In such organoid morphology, budded crypt-like structures represent intestinal stem-cell enriched domains that give rise to differentiated cells that populate the main body of the organoid (Oost et al., 2018; Rodriguez-Colman et al., 2017). As expected, transcript expression of the differentiated cell marker *KRT20* was largely confined to the main body of the organoid, while *LGR5^+^* stem cells were relatively enriched within budding crypts (Fig. 3E,F). These findings thus indicate that the smFISH method described here allows for a faithful evaluation of the spatial distribution of stem- and differentiated-cell domains within 3D cultured tissues.

### Concluding remarks

Here we present a method for smFISH-based detection of transcripts in 3D in whole human organoids. By employing dual fluorescence to target a single transcript we provide an easily applicable tool for method optimization and validation. Our protocol further allows for the preservation of 3D organoid morphology and a sensitive and specific detection of alterations in transcript abundance upon perturbation of signaling pathways involved in adult tissue homeostasis.

Multiple organoid culture systems for a variety of epithelial tissues were established recently, including liver (Huch et al., 2015), pancreas (Huch et al., 2013), stomach (Stange et al., 2013), prostate (Drost et al., 2016), esophagus (DeWard et al., 2014), fallopian tube (Kessler et al., 2015), taste buds (Ren et al., 2014), salivary glands and tongue (Maimets et al., 2016). We envision that our method can be applied easily to other organoid systems with no or minimal optimization.

## MATERIALS AND METHODS

### Cell culture, plasmids and transfection

SW480 cells from ATCC were grown in DMEM (Lonza; 4.5 g glucose/l) supplemented with 10% FBS (Bodinco B.V.) and penicillin/streptomycin (Sigma-Aldrich; 50 μg/ml). All cells were grown at 37°C and 5% CO_2_. SP-dCas9-VP64-p65-Rta (VPR) (Plasmid #63798) and EF1a-dCas9-KRAB-T2A-Puro-WPRE (Plasmid #99372) were obtained from Addgene. Four guide RNAs, targeting human *APC* promoter region, were cloned in gRNA concatemer vector as previously described (Andersson-Rolf et al., 2016). *APC* gRNAs were designed with sgRNA design tool (http://sam.genome-engineering.org/). *APC* gRNA concatemer and dCas9-VPR or dCas9-KRAB plasmids were co-transfected by using Lipofectamine 2000 (Invitrogen) according to the manufacturer's instructions. For Wnt3a-conditioned medium (Wnt3a-CM), mouse L-cells were cultured in DMEM containing 1 g/l glucose (Thermo Fisher Scientific) supplemented with 10% Fetal Calf Serum (Bodinco B.V.), 100 units/ml penicillin and 100 μg/ml streptomycin (Thermo Fisher Scientific). L-cells stably expressing Wnt3a were used to generate Wnt3a-CM as described previously (Tauriello et al., 2010). All cells were grown at 37°C at 5% CO_2_. Mycoplasma contamination was tested for monthly and was always negative.

### Organoid handling

All experimentation using human organoids described herein was approved by the ethical committee at University Medical Center Utrecht (UMCU; TcBio #12-093). Informed consent for tissue collection, generation, storage and use of the organoids was obtained from the patients at UMCU. Human healthy colon organoids were cultured as described previously (van de Wetering et al., 2015). Organoid culture medium contained advanced DMEM/F12 medium (Invitrogen) supplemented with penicillin/streptomycin (Sigma-Aldrich), 10 mM HEPES (Life Technologies), 1x Glutamax (Life Technologies) (defined as AdvDMEM^+/+/+^) including 1X B27 (Invitrogen), 10 mM nicotinamide (Sigma-Aldrich), 1.25 mM N-acetylcysteine (Sigma-Aldrich), Noggin-conditioned medium (10% v/v), R-spondin1-conditioned medium (20% final volume if not indicated otherwise), 50 ng/ml EGF (Peprotech), WNT3a conditioned medium (50% final volume if not indicated otherwise, produced using stably transfected L cells), 500 nM TGF-β type I receptor inhibitor A83-01 (Tocris) and 10 µM P38 inhibitor SB202190 (Sigma-Aldrich). IWP-2 (R&D systems) was used at 2.5 μM for 2 days.

### Preparation of organoids for smFISH

Organoids were passaged two days before applying smFISH. Specifically, culture medium was removed and ice-cold AdvDMEM^+/+/+^ was added to each well. Matrigel was disrupted by pipetting with a glass Pasteur pipet several times. Next, organoids were further dissociated by pipetting (∼10×) with a plastic cooled syringe. Finally, organoids were seeded in Matrigel (Corning)/AdvDMEM^+/+/+^ in a ratio 1:1. After two days, one to two wells from a 24-well plate of high confluence organoids were harvested in ice-cold AdvDMEM^+/+/+^ and washed three times with 10 ml of ice-cold AdvDMEM^+/+/+^ per each smFISH conditions. Organoids were pelleted by centrifugation (3 min, 50×***g***, cooled centrifuge). Cell Recovery Solution (Corning) was applied and organoids were re-suspended five times and left for 15 min on ice. Organoids were then washed three times with ice-cold PBS solution (3 min, 50×g, cooled centrifuge) and re-suspended in 10 μl of ice-cold PBS solution.

### Probe design

Experiment were performed with custom made smFISH probes designed by Stellaris (Biosearch Technologies). All the probes were directed against the coding region of the transcripts by using Probe Designer Tool (http://www.biosearchtech.com/stellarisdesigner/). *APC* probe sets were labeled with Quasar 670 or TAMRA fluorophores. *LGR5* probes were labeled with Quasar 670, *KRT20* probes with Quasar 570 and *AXIN2* probes with Quasar 670.

### Spotting organoids on the coverslips and smFISH

20×20 mm coverslips (Sigma-Aldrich) were coated with Poly-l-lysine hydrobromide (Sigma-Aldrich) solution for 1 h, dipped twice in RNAse clean water and dried. After washing, Matrigel-free organoids were collected as a pellet in 15 ml falcon tubes. Pellets were re-suspended in 10 μl RNAse-free PBS (Ambion) and spotted as a droplet on coated coverslips. By using the pipet tip, the droplet was spread over the coverslip and residual PBS was removed. The coverslips (with organoids) were put in a six-well plate and placed on dry ice for 5 min. Afterwards, organoids were fixed and hybridized as previously described (Lyubimova et al., 2013). Briefly, organoids were fixed with 2 ml of fixation buffer (4% Formaldehyde; Sigma-Aldrich) for 10 min. Next, coverslips were washed 2X with RNAse-free PBS and permeabilized with 70% Ethanol overnight at 4°C. The next day, coverslips were washed with 10% smFISH washing buffer [1X saline-sodium citrate (20X stock; Sigma-Aldrich), 10% Formamide (Ambion)] and placed with organoids facing down on a 100 μl droplet of hybridization buffer [7.3 ml H_2_O, 1 g of dextran sulfate (Sigma-Aldrich), 1 ml 20X saline-sodium citrate (Sigma-Aldrich), 10% Formamide (Ambion), 500 μl of tRNA stock (20 mg/ml Roche), 40 μl of BSA stock (50 mg/ml, Roche), 100 μl of Ribonucleoside Vanadyl Complex (200 mM, New England Biolabs)], containing 1 μl of smFISH probe stock (Stellaris). Hybridization was performed at 37°C for 4 h. Next, coverslips were placed back in the six-well plate (organoids facing up) and washed with 2 ml of 20% smFISH washing buffer [1X saline-sodium citrate (20X stock; Sigma-Aldrich), 20% Formamide (Ambion)] for 30 min at 37°C. This step was repeated again with fresh 20% smFISH washing buffer containing Hoechst (Thermo Fisher Scientific) for nuclei counterstain. After washing, the coverslips were equilibrated in 2 ml GLOX buffer [8.5 ml H20, 100 μl Tris (1M, pH 8.0; Ambion), 1 ml of 20X saline-sodium citrate (Sigma-Aldrich), 400 μl of 10% Glucose (Sigma-Aldrich)] for 5 min.

### Imaging

Imaging of organoids was performed by mounting coverslips with organoids on objective glasses using rubber cement (Fixo Gum). Imaging buffer consisted of GLOX buffer supplemented with Glucose Oxidase (Sigma-Aldrich) and Catalase (Sigma-Aldrich) and was prepared as previously described (Lyubimova et al., 2013). All images were acquired on a deconvolution system (DeltaVision RT; Applied Precision) using a 60x objective (Applied Precision). Z-stack images were acquired in order to obtain whole organoids, usually spanning 10–18 μm in size. All images of simultaneously stained experiments were acquired with identical illumination settings. Deconvolved stacks were used for quantification.

### RT-qPCR

Organoids were seeded in a 12-well plate (one well per condition) four days before RNA isolation. After 48 h, the medium was replaced by either full medium, Wnt3a and R-spondin reduced (25% and 10%, respectively) or 2.5 μM of IWP-2 supplemented medium for 48 h. Next, RNA was isolated using RNeasy (Qiagen), followed by DNAse treatment (Invitrogen). RT-qPCR of three independent experiments was done in duplicate. Primers used: AAAGAGAGGAGGTTCAGATG (*AXIN2* Forward); CTGAGTCTGGGAATTTTTCTTC (*AXIN2* Reverse); GGAATGTGTCCAGCTTGATA (*APC* Forward); CACAAAGTTCCACATGCATT (*APC* Reverse).

### smFISH in cells

Cells were grown on coverslips in the presence of *APC* gRNA and dCas9-VPR or dCas9-KRAB plasmids for 48 h. Afterwards, cells were fixed for 10 min with 4% Formaldehyde solution (Sigma-Aldrich) and 70% Ethanol overnight. For the smFISH, the samples were prepared as previously described (Raj and Tyagi, 2010). Briefly, samples were hybridized with *APC* probes (Stellaris, Biosearch Technologies) for 4 h at 37°C and mounted to the microscopy slide using Prolong Diamond Antifade (Invitrogen). The images were acquired on a deconvolution system (DeltaVision RT; Applied Precision) using 60× lens.

### Data analysis

Numbers of *APC* mRNA per cell were calculated by dividing the number of *APC* mRNA over total number of nuclei, as measured in each individual ROI. Counting the number of Hoechst stained nuclei was performed manually, *APC* mRNA number was measured by MATLAB code. Our algorithm, which is based on the MATLAB implementation of (Raj et al., 2008), locates mRNA dots in two channels and determines whether dots between the two channels co-localize. To locate the mRNA dots, the raw image stack is binarized using a channel specific threshold. This channel specific threshold is selected by plotting the amount of detected molecules as a function of the binarization threshold. The final threshold is chosen to coincide with the inflection point of the curve. Connected components are obtained from the binarized cube. We assume that every connected component corresponds to a single spot. Noisy connected components are filtered by thresholding for minimum and maximum dot volume. Co-localization is determined based on the pairwise Euclidean distance between the centroids of the connected components. Dots are considered to co-localize when the distance between centroids is below the distance threshold of 3 voxels. To prevent overestimating the amount of co-localizing molecules, every dot can be paired to at most one dot in the other channel. Our code is available through Github at https://github.com/UMCUGenetics/smFISH-organoids.

### Statistics and reproducibility

Results were obtained by at least three independent biological replicates. For statistical analysis, we confirmed Gaussian distribution of data using D'Agostino-Pearson normality test. To test significance, we used ordinary one-way analysis of variance (ANOVA) with Tukey's multiple comparisons test as follow-up (with 95% confidence interval). In Figs 2F and 3F, Fig. S1D and S1F an unpaired Student's *t*-test was performed. Data are depicted as mean±s.e. of the mean (s.e.m.). Calculations were made using GraphPad Prism software.

## Supplementary Material

Supplementary information
