# Supplementary material for: Three-dimensional analysis of single molecule FISH in human colon organoids
Source: Biol Open. 2019 Jul 30;8(8):bio042812. doi: 10.1242/bio.042812 (PMC6737975; doi:10.1242/bio.042812)
Supplement: Supplementary information [file biolopen-8-042812-s1.pdf]

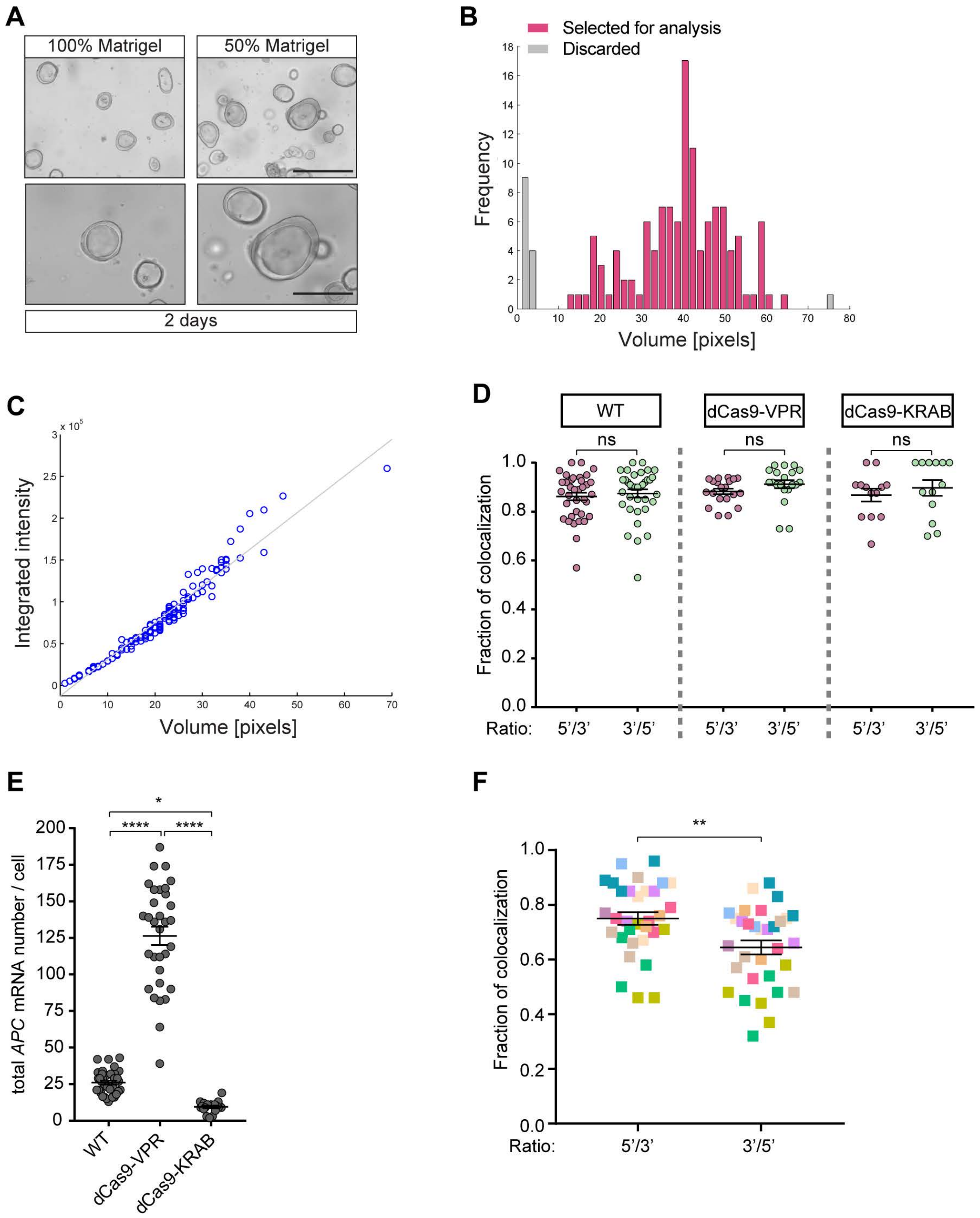

Biology Open • Supplementary information

Figure S1: Validation of *APC* smFISH probes in 2D culture

(A) Bright field microscopy of human colon organoids after 2 days of culturing in 100% Matrigel of 50% Matrigel. Scale bars represent 400 μm (upper panel) or 200 μm (lower panel). (B) Graph represents the distribution of mRNA dots after applying thresholding. Pink bars represent selected dots and gray bars discarded dots. (C) The relation between the summed fluorescence intensity and volume of the dots is linear. (D) *APC* smFISH probe co-localization in wild type (WT; n=36), dCas9-VPR (n=32) or dCas9-KRAB (n=13) transfected SW480 cells. Each dot represents one cell. *APC* Quasar 670- (5') and TAMRA-labelled (3') probes were used. Data of three independent experiments are shown (± S.E.M.). To test significance, we used an unpaired *t*-test. Ns = not significant. (E) Total number of *APC* mRNA transcripts as measured in wild type (WT; n=36), dCas9-VPR (n=32) or dCas9-KRAB (n=13) transfected SW480 cells. Single cells were analyzed by MATLAB code. Data of three independent experiments are shown (± S.E.M.). Each dot represents one cell. To test significance, ordinary one-way ANOVA was used. Ns = not significant. *P=0.0262, ****P<0.0001. (F) Longer treatment (60 minutes incubation) with Cell Recovery influences smFISH signal, as seen by overall lower co-localization levels and high variation between different ROIs. Organoids are color-coded, each color indicates individual ROI within a single organoid. Analyzed organoids in both conditions n=10. Data of three independent experiments are shown (± S.E.M.).To test significance, an unpaired *t*-test was used. **P=0.0033.

Biology Open • Supplementary information

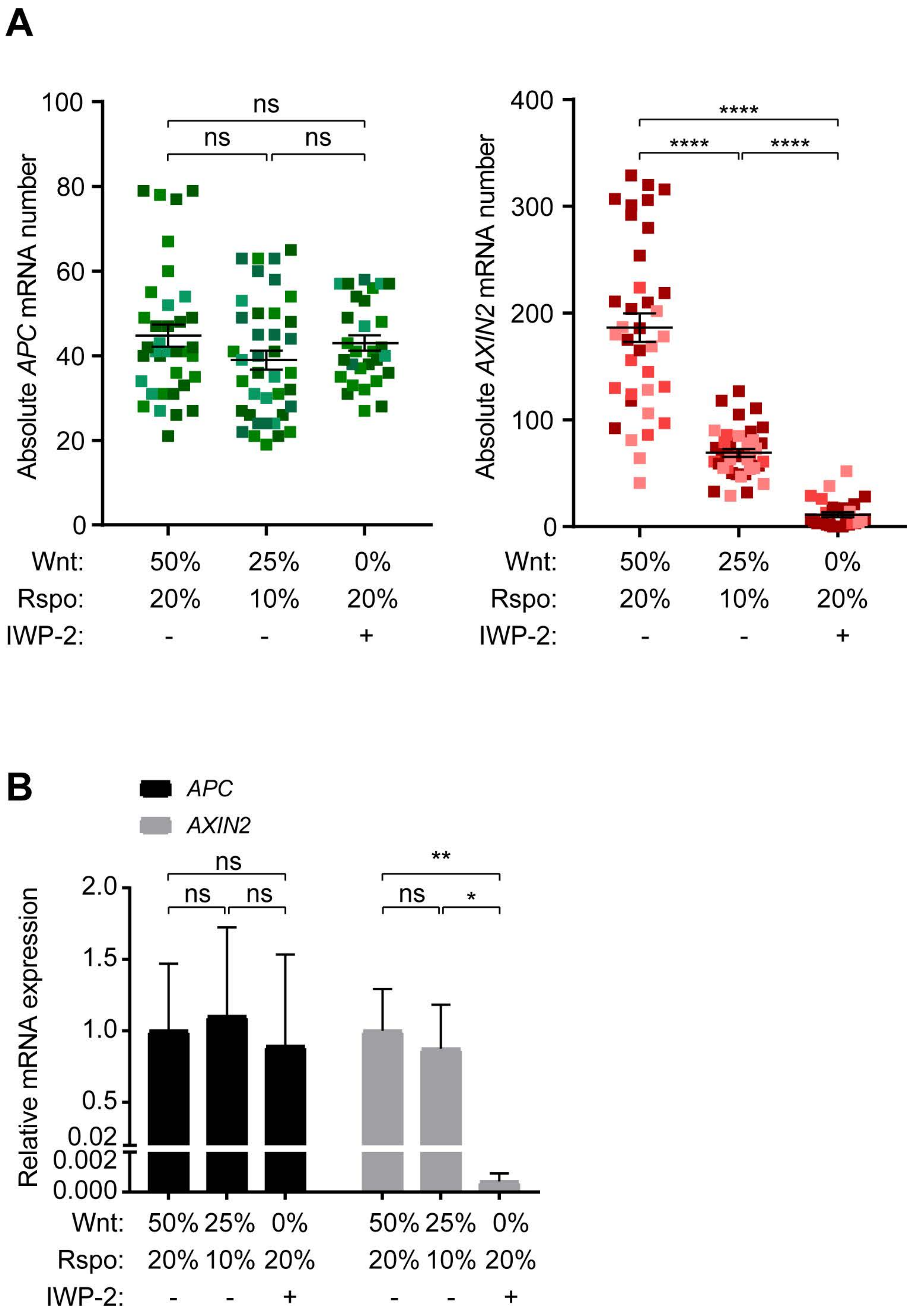

Biology Open • Supplementary information

Figure S2: *APC* and *AXIN2* smFISH signals are qualitative and quantitative

(A) Graphs show absolute *APC* or *AXIN2* mRNA number in human colon organoids, as measures by smFISH. Organoids were grown in full medium (50% Wnt and 20% Rspo), Wnt3a and R-spondin reduced (20% and 10%, respectively) or IWP-2 supplemented medium for 48h, as indicated. One square represents one ROI. Full medium, n=19; Wnt3a and R-spondin reduced n=18; IWP-2 n=16. Data of three independent experiments are shown (± S.E.M), represented by different shades of green (*APC* smFISH) or red (*AXIN2* smFISH). To test significance, ordinary one-way ANOVA was used. Ns = not significant; ****P<0.0001. (B) RT-qPCR of human colon organoids, treated as indicated. Data were normalized to full medium and calculated as 2^-ΔCt. Data of three independent experiments are shown (±S.E.M.). To test significance, ordinary one-way ANOVA was used. Ns = not significant; *P =0.0113; **P = 0.0060.
